# MgrA Negatively Regulates Biofilm Formation and Detachment by Repressing the Expression of *psm* Operons in Staphylococcus aureus

**DOI:** 10.1128/AEM.01008-18

**Published:** 2018-08-01

**Authors:** Qiu Jiang, Zeyu Jin, Baolin Sun

**Affiliations:** a Hefei National Laboratory for Physical Sciences at Microscale, CAS Key Laboratory of Innate Immunity and Chronic Disease, School of Life Sciences, University of Science and Technology of China, Hefei, Anhui, China; University of Tokyo

**Keywords:** Staphylococcus aureus, phenol-soluble modulins, MgrA, biofilm

## Abstract

Phenol-soluble modulins (PSMs) are amphipathic peptides that are produced by staphylococci and play important roles in Staphylococcus aureus biofilm formation and dissemination. Although the multiple functions of PSMs have been recognized, the regulatory mechanisms controlling the expression of *psm* operons remain largely unknown. In this study, we identified MgrA in a DNA pulldown assay and further demonstrated, by electrophoretic mobility shift assays and DNase I footprinting assays, that MgrA could bind specifically to the promoter regions of *psm* operons. We then constructed an isogenic *mgrA* deletion strain and compared biofilm formation and detachment in the wild-type and isogenic *mgrA* deletion strains. Our results indicated that biofilm formation and detachment were significantly increased in the *mgrA* mutant strain. Real-time quantitative reverse transcription-PCR data indicated that MgrA repressed the transcription of *psm* operons in cultures and biofilms, suggesting that MgrA is a negative regulator of *psm* expression. Furthermore, we analyzed biofilm formation by the *psm* mutant strains, and we found that PSMs promoted biofilm structuring and development in the *mgrA* mutant strain. These findings reveal that MgrA negatively regulates biofilm formation and detachment by repressing the expression of *psm* operons through direct binding to the *psm* promoter regions.

**IMPORTANCE** Staphylococcus aureus is a human and animal pathogen that can cause biofilm-associated infections. PSMs have multiple functions in biofilm development and virulence in staphylococcal pathogenesis. This study has revealed that MgrA can negatively regulate *psm* expression by binding directly to the promoter regions of *psm* operons. Furthermore, our results show that MgrA can modulate biofilm structuring and development by repressing the production of PSMs in S. aureus. Our findings provide novel insights into the regulatory mechanisms of S. aureus psm gene expression, biofilm development, and pathogenesis.

## Abstract (precis)

Staphylococcus aureus is a human and animal pathogen that can cause biofilm-associated infections. PSMs have multiple functions in biofilm development and virulence in staphylococcal pathogenesis. This study has revealed that MgrA can negatively regulate *psm* expression by binding directly to the promoter regions of *psm* operons. Furthermore, our results show that MgrA can modulate biofilm structuring and development by repressing the production of PSMs in S. aureus. Our findings provide novel insights into the regulatory mechanisms of S. aureus psm gene expression, biofilm development, and pathogenesis.

## INTRODUCTION

Staphylococcus aureus is a human and animal pathogen that can cause various bacterial infections, including relatively benign and fatal systemic diseases (1, 2). S. aureus can develop biofilms on host tissues and medical devices and can cause chronic infections when S. aureus cells accumulate to form biofilms at the infection sites (3). Biofilms represent a protected environment that is essential for bacteria to resist host immune responses (4) and chemotherapies (5), making therapeutic intervention extremely difficult. Moreover, biofilm dissemination enables bacteria to spread, leading to infections in the body (6–8).

Biofilms are known as multicellular structures encased in a matrix of proteins, polysaccharides, extracellular DNA (eDNA), and other environmental factors (9). Biofilm formation is modulated by various regulatory systems, including the *agr* quorum-sensing system (10), the LuxS/AI-2 quorum-sensing system (11), the ArlR/S two-component system (12), and transcriptional regulators, such as MgrA (13), Rbf (14), Sar-family proteins (15–17), and IcaR (18). These regulators modulate biofilm formation by regulating the production of biofilm-formation-associated factors, including surface proteins, polysaccharide intercellular adhesin (PIA), eDNA, and other extracellular components in biofilms. For instance, MgrA can repress biofilm formation by controlling the release of eDNA and the expression of surface proteins and extracellular proteases in S. aureus (13, 19). The *agr* quorum-sensing system controls biofilm detachment by regulating biofilm-detachment-associated factors, including extracellular proteases, nucleases, and phenol-soluble modulins (PSMs) (10, 20). Biofilms are complex, however, and the regulatory mechanisms controlling biofilm development in S. aureus have not been thoroughly elucidated.

PSMs are known as biofilm-structuring-associated and biofilm-dissemination-associated factors involved in S. aureus biofilm-associated infections (8, 21–23). PSMs are amphipathic and surfactant-like peptides containing five α-peptides (PSMα1 to -4 and δ-toxin) and two β-peptides (PSMβ1 and -2) (24). PSMαs are encoded by the *psmα* operon, PSMβs are encoded by the *psmβ* operon, and the δ-toxin (also called δ-hemolysin [Hld]) is encoded within RNAIII, regulatory RNA encoded from the *agr* operon (24–26). With the given surfactant-like characteristics, PSMs promote biofilm structuring to form channels that make nutrition available to bacteria and facilitate biofilm detachment to free bacteria (21). Although the functions of PSMs have been studied extensively, the detailed regulatory mechanisms controlling the production of PSMs in S. aureus are poorly defined.

In this study, we found that MgrA could specifically bind to the promoter regions of *psm* operons and negatively regulate the expression of *psm* genes in S. aureus NCTC8325. Our findings reveal a regulatory mechanism through which MgrA regulates the production of PSMs to modulate biofilm formation and detachment in S. aureus.

## RESULTS

### Proteins binding the *psmα* promoter are identified.

**FIG 1:**
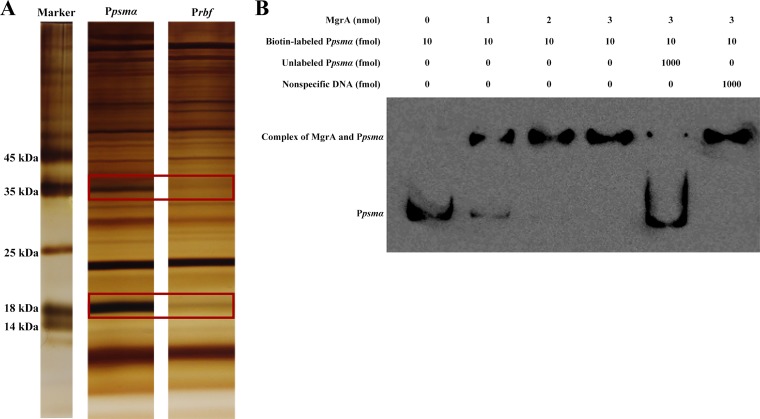
Identification of the *psmα* promoter-specific binding protein. (A) SDS-PAGE analysis of proteins binding to the *psmα* promoter region or the *rbf* promoter region. The gel was stained with silver, and the specific bands boxed in red were analyzed by LC-MS/MS. (B) EMSA of the purified MgrA with a 336-bp *psmα* promoter fragment labeled with biotin. Increasing concentrations of the purified MgrA protein and 10 fmol of the P*psmα*-biotin probe were used in the reactions, and the reaction mixtures were incubated at 25°C for 1 h. The specific competition probe was the unlabeled *psmα* promoter fragment, and a *pta* gene fragment was used as a nonspecific competition probe. P*psmα*, *psmα* promoter fragment; P*psmα*-biotin, biotin-labeled P*psmα*; P*rbf*, *rbf* promoter fragment.

Previous studies have shown that PSMs are involved in the biofilm development and virulence of S. aureus (20, 21). AgrA is a positive regulator of *psm* operons and strictly regulates *psm* transcription in S. aureus (20). However, regulators other than AgrA that can directly regulate *psm* expression in S. aureus have not been reported. To identify other transcriptional factors that can directly regulate the expression of *psm* genes, we performed DNA affinity pulldown assays to screen for DNA-binding proteins in S. aureus. A biotin-labeled DNA fragment containing the promoter region of the *psmα* operon was amplified with primers P*psmα*-F and P*psmα*-biotin-R and then used for DNA pulldown assays. The pulled-down proteins were subjected to SDS-PAGE and liquid chromatography-tandem mass spectrometry (LC-MS/MS) analyses, as described in Materials and Methods. Potential DNA-binding proteins were separated by SDS-PAGE and stained with silver. The proteins that bound specifically to the promoter region of the *psmα* operon were identified by LC-MS/MS. The DNA-binding proteins are listed in Table 1, and proteins that did not contain DNA-binding domains are listed in Table S1 in the supplemental material. MgrA (∼17 kDa) is a helix-turn-helix (HTH)-type transcriptional regulator involved in autolytic activity (27), multidrug resistance (28), and virulence (29), suggesting that MgrA could be a regulator of the *psmα* operon. The hypothetical protein SAOUHSC_03049 (∼32 kDa) is an uncharacterized protein that contains a DNA-binding domain and belongs to the ParB family, which is involved in chromosome partitioning and cell division processes (30). It has been reported that *SAOUHSC_03049* is an essential gene and its main function is similar to that of *yyaA* (also called *noc*), affecting nucleoid occlusion by binding nonspecifically to DNA in Bacillus subtilis (30, 31). Considering the function of the hypothetical protein SAOUHSC_03049 in chromosome partitioning, we did not choose it for further study, although it could be a transcriptional regulator in S. aureus. Other proteins, including PolA, TopA, RuvA, GyrA, and RpoC, are general DNA-binding proteins related with DNA duplication or repair. Two bands (∼18 kDa and 35 kDa) were specific to the promoter region of the *psmα* operon, compared with the control (Fig. 1A). Based on Table 1 and Table S1 in the supplemental material, the apparent 18-kDa protein band was most likely MgrA. The apparent 35-kDa protein band seemed to contain several proteins (Table 2). Among those proteins, only the hypothetical protein SAOUHSC_03049 (∼32 kDa) contained a DNA-binding domain, as described above (Tables 1 and 2). In this study, we aimed to identify the main transcriptional factors that regulate the expression of *psm* genes by binding directly to the promoter region of *psm* operons. Thus, only MgrA, among those pulled-down proteins, was considered a regulator of the *psmα* operon for further study.

**TABLE 1:** Proteins with DNA-binding domains identified by LC-MS/MS

Gene	Protein	Molecular function
SAOUHSC_00694	MgrA	HTH-type transcriptional regulator MgrA; DNA binding transcription factor activity; transcription regulatory region DNA binding
SAOUHSC_03049	Hypothetical protein SAOUHSC_03049	Uncharacterized protein; DNA binding; similar to ParB, probably involved in chromosome partitioning and cell division processes
SAOUHSC_01797	PolA	DNA polymerase I; 3′-5′ exonuclease activity; 5′-3′ exonuclease activity; DNA binding; DNA-directed DNA polymerase activity
SAOUHSC_01222	TopA	DNA topoisomerase 1; DNA binding; DNA topoisomerase type I activity; metal ion binding
SAOUHSC_01751	RuvA	Holliday junction ATP-dependent DNA helicase; ATP binding; DNA binding; four-way junction helicase activity
SAOUHSC_00006	GyrA	DNA gyrase subunit A; ATP binding; DNA binding; DNA topoisomerase type II (ATP-hydrolyzing) activity
SAOUHSC_00525	RpoC	DNA-directed RNA polymerase subunit β′; DNA binding; DNA-directed 5′-3′ RNA polymerase activity

**TABLE 2:** Proteins with estimated molecular masses of 32 to 36 kDa identified by LC-MS/MS

Gene	Protein	Molecular mass (Da)	Molecular function
SAOUHSC_03049	Hypothetical protein SAOUHSC_03049	32,197	Uncharacterized protein; DNA binding; similar to ParB, probably involved in chromosome partitioning and cell division processes
SAOUHSC_01041	Pyruvate dehydrogenase complex, E1 component subunit β	35,246	Pyruvate dehydrogenase complex, putative E1 component β subunit; pyruvate dehydrogenase (acetyl group-transferring) activity
SAOUHSC_00472	RPPK	35,284	Ribose phosphate pyrophosphokinase; ATP binding; kinase activity; magnesium ion binding; ribose phosphate diphosphokinase activity
SAOUHSC_00499	PdxS	31,993	Pyridoxal 5′-phosphate synthase subunit PdxS; pyridoxal 5′-phosphate synthase (glutamine-hydrolyzing) activity
SAOUHSC_00206	L-LDH-1	34583	l-Lactate dehydrogenase 1; l-lactate dehydrogenase activity

### MgrA specifically binds to the promoter region of the *psmα* operon.

MgrA (also called Rat or NorR), a homolog of MarR and SarA, contains a DNA-binding HTH motif and regulates certain target genes in S. aureus by binding directly to their promoter regions (16, 17, 19, 29). To confirm its ability and specificity to bind to the promoter region of the *psmα* operon *in vitro*, we purified a 6His-tagged MgrA protein and employed the same 336-bp *psmα* operon promoter fragment used in the DNA affinity pulldown assay to perform electrophoretic mobility shift assays (EMSAs). DNA fragments (10 fmol per reaction mixture) were end-labeled with biotin and used in EMSAs with various amounts of purified MgrA. MgrA was able to bind to the *psmα* promoter region (Fig. 1B). This binding could be outcompeted with a 100-fold excess of an identical unlabeled *psmα* promoter DNA fragment, while a 100-fold excess of a nonspecific probe was not able to compete for MgrA binding. This result further suggested that MgrA could specifically bind to the promoter region of the *psmα* operon.

### The recognition region of MgrA on the *psmα* promoter overlaps the −35 and −10 regions.

**FIG 2:**
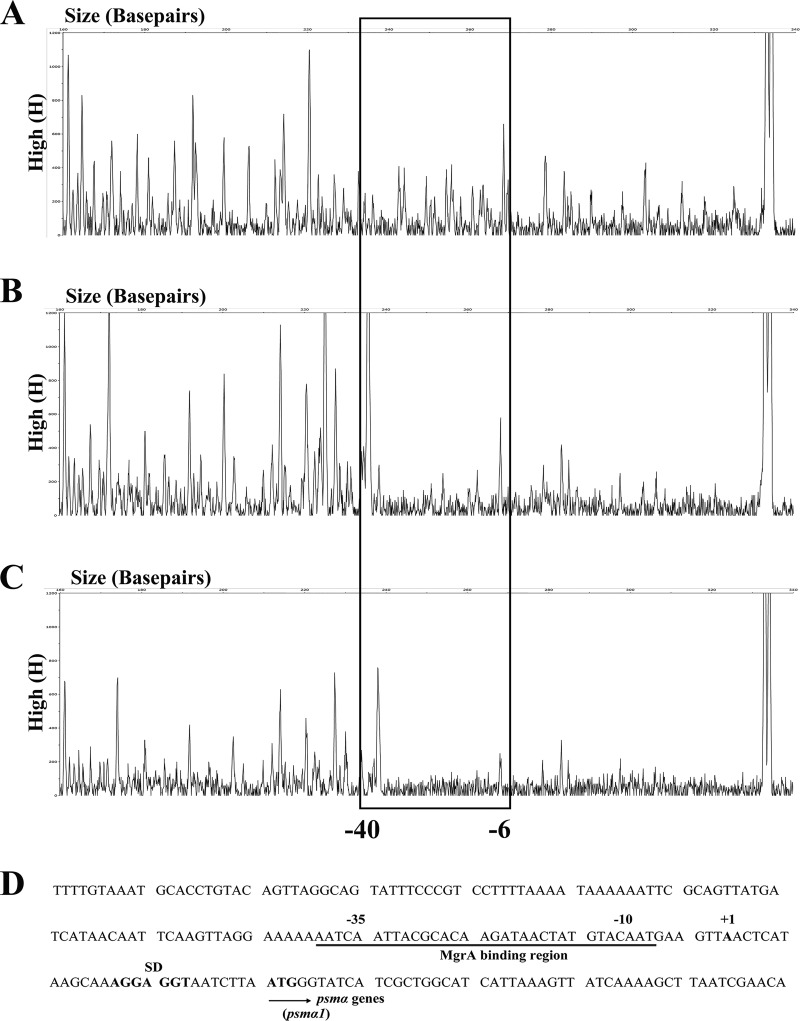
(A to C) Mapping of the MgrA recognition site in the *psmα* promoter by DNase I footprinting. The 336-bp *psmα* promoter fragment was labeled with 6-FAM, and probes (1 pmol/100 μl) were incubated for 1 h at 25°C with MgrA at 0 μg/100 μl (A), 2 μg/100 μl (B), or 4 μg/100 μl (C) and then digested for 1 min at 37°C with DNase I at 0.1 U/100 μl. The protected region of MgrA is boxed in black. (D) MgrA binding sequence in the *psmα* promoter region. The MgrA binding region, based on the DNase footprinting analyses, is underlined in black. SD, Shine-Dalgarno sequence.

Since MgrA could bind directly to the promoter region of the *psm*α operon, we were interested in the MgrA recognition sites on the *psmα* operon promoter. We performed DNase I protection footprinting assays with the same 336-bp *psmα* operon promoter fragment labeled with 6-carboxylfluorescein (6-FAM) (Fig. 2). The 6-FAM-labeled DNA fragment was evenly digested by DNase I when MgrA protein was not added, as reflected by the even distribution of the 6-FAM signals (Fig. 2A). The region from −6 to −40 bp, relative to the transcription start site of the *psmα* operon (20), was protected, as indicated by the disappearing nucleotide peaks in Fig. 2A, compared to those in Fig. 2B and C. These data indicated that the MgrA recognition site may lie in the 34-bp region (AAATCAATTACGCACAAGATAACTATGTACAATG) of the *psmα* promoter overlapping the −35 to −10 transcriptional boxes of the *psmα* operon (Fig. 2D).

### MgrA is a negative regulator of *psm* operons.

**FIG 3:**
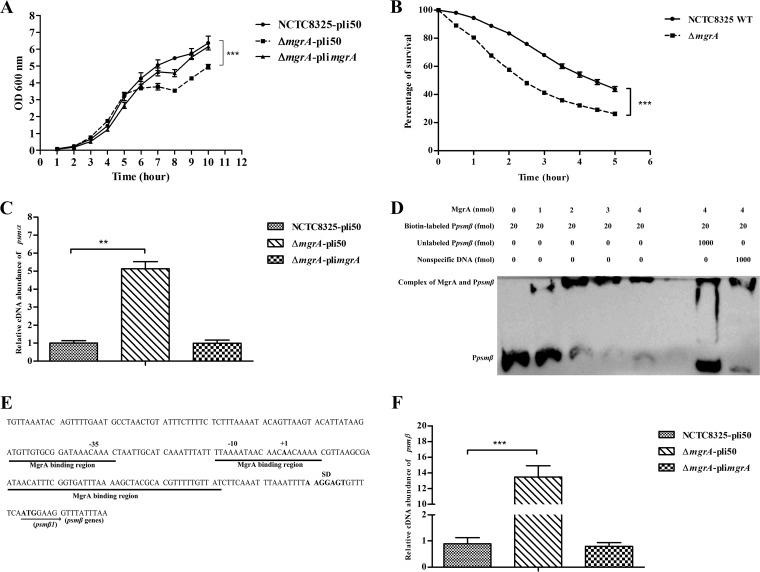
MgrA as a negative regulator of *psm* operons. (A) Growth curves of NCTC8325-pli50, Δ*mgrA*-pli50, and Δ*mgrA*-pli*mgrA* strains. Bacteria were grown at 37°C in TSB containing 15 μg/ml Cm, with shaking. OD_600_ values were measured every 1 h. Values are from three biological replicates (mean ± standard error of the mean [SEM]). Statistical values were determined with the Student *t* test and the F test to compare variances. ***, *P* < 0.001. (B) Autolysis induced by Triton X-100. Bacteria were grown at 37°C in TSB, with shaking, and cultures were collected at an OD_600_ of 3. Triton X-100 (0.1%) in Tris buffer (pH 7.5) was used to induce bacterial autolysis. Values are from three biological replicates (mean ± SEM). Statistical values were determined with the Student *t* test and the F test to compare variances. ***, *P* < 0.001. (C) qRT-PCR of *psmα* in NCTC8325-pli50, Δ*mgrA*-pli50, and Δ*mgrA*-pli*mgrA* strains. Bacteria were grown at 37°C in TSB, with shaking. The cDNA samples used were prepared from RNA isolated from cells grown to the indicated growth phases (10 h). Probes were designed to align with part of the *psmα* operon. Values are from three biological replicates (mean ± SEM). Statistical values were determined by one-way analysis of variance (ANOVA). **, *P* < 0.01. (D) EMSA of purified MgrA with a 243-bp *psmβ* promoter fragment labeled with biotin. Increasing concentrations of the purified MgrA protein and 20 fmol of the P*psmβ*-biotin probe were used in the reactions, and the reaction mixtures were incubated at 25°C for 1 h. The specific competition probe was the unlabeled *psmβ* promoter fragment, and a *pta* gene fragment was used as a nonspecific competition probe. (E) Putative MgrA-binding regions (underlined in black), based on the sequence analysis. (F) qRT-PCR of *psmβ* in NCTC8325-pli50, Δ*mgrA*-pli50, and Δ*mgrA*-pli*mgrA* strains. The cDNA samples used were prepared from RNA isolated from cells grown to the indicated growth phase (10 h). Probes were designed to align with part of the *psmβ* operon. Values are from three biological replicates (mean ± SEM). Statistical values were determined by one-way ANOVA. ***, *P* < 0.001. NCTC8325-pli50, NCTC8325 WT strain carrying plasmid pli50; Δ*mgrA*-pli50, *mgrA* mutant strain carrying plasmid pli50; Δ*mgrA*-pli*mgrA*, *mgrA* mutant strain carrying plasmid pli*mgrA*; P*psmβ*, *psmβ* promoter fragment; P*psmβ*-biotin, biotin-labeled P*psmβ*; SD, Shine-Dalgarno sequence.

We constructed the isogenic *mgrA* deletion and *mgrA*-complemented strains and determined the transcriptional levels of the *psmα* operon in the wild-type (WT), *mgrA* mutant, and *mgrA*-complemented strains. First, we determined the growth curves of these strains and found that the *mgrA* mutant strain exhibited a nongrowth state at the 6- to 8-h time point, compared with the WT strain (Fig. 3A). Previous studies demonstrated that the autolysis of the *mgrA* mutant strain was increased at the mid-exponential phase (27), and the similar result was observed in our study (Fig. 3B). Real-time quantitative reverse transcription-PCR **(**qRT-PCR) was performed to determine the transcriptional levels of the *psmα* operon. The transcriptional level of *psmα* was significantly increased in the *mgrA* mutant strain, compared with the WT strain, and the change could be reversed by introducing pli*mgrA* into the *mgrA* mutant strain (Fig. 3C). These data further indicated that MgrA could act as a negative regulator to modulate the expression of the *psmα* operon in S. aureus.

Unlike PSMα, PSMβ is expressed from the *psmβ* operon (24). We further determined whether MgrA could regulate the expression of the *psmβ* operon in S. aureus. To determine whether MgrA could bind to the promoter region of the *psmβ* operon, we employed a 243-bp *psmβ* promoter fragment upstream of the initiation codon (20) to perform an EMSA. MgrA was able to bind to the promoter region of the *psmβ* operon; this binding could be outcompeted with a 50-fold excess of an identical unlabeled *psmβ* promoter DNA fragment, and a 50-fold excess of a nonspecific probe was not able to outcompete MgrA binding (Fig. 3D). The DNase I footprinting assay was performed with the same 243-bp promoter fragment of the *psmβ* operon labeled with 6-FAM, but it failed to identify the MgrA-binding region. Then, we analyzed the promoter sequence of the *psmβ* operon and found that several putative MgrA-binding regions may exist (Fig. 3E). Furthermore, qRT-PCR was performed to determine the transcriptional levels of *psmβ* in the WT, *mgrA* mutant, and *mgrA*-complemented strains. The transcriptional level of *psmβ* was significantly increased in the *mgrA* mutant strain, and the change could be reversed by introducing pli*mgrA* into the *mgrA* mutant strain (Fig. 3F). These data indicated that MgrA could repress the expression of the *psmβ* operon by binding directly to the promoter region of the *psmβ* operon.

The amounts of PSMs in cultures can be determined by reverse-phase (RP)-high-performance liquid chromatography (HPLC) (24). Since MgrA can repress the expression of the *psmα* and *psmβ* operons, we analyzed the production of PSMs by RP-ultra-performance liquid chromatography (UPLC) to determine the changes of PSMs in the *mgrA* mutant strain. Overnight cultures were collected, and the culture filtrate samples were analyzed by RP-UPLC. The signals of PSMs were significantly enhanced in the *mgrA* mutant strain, compared with the WT strain (Fig. S1). These results suggested that MgrA could repress the transcription of *psm* operons and the repression led to the alteration of PSM production.

### MgrA regulates biofilm development by repressing the expression of *psm* operons.

**FIG 4:**
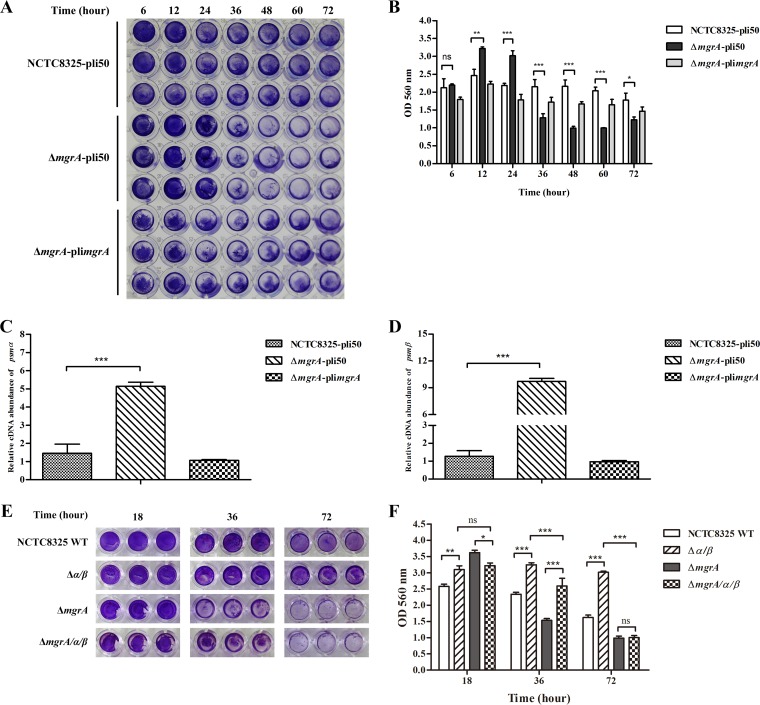
MgrA modulation of biofilm development by repression of *psm* gene expression. (A) Biofilm formation by NCTC8325-pli50, Δ*mgrA*-pli50, and Δ*mgrA*-pli*mgrA* strains. Static biofilms were grown in 96-well plates at 37°C. At the indicated time points, biofilms were washed, stained with 0.5% crystal violet, and visualized with a camera. (B) Biofilm quantitation. Stained biofilms (A) were solubilized with 30% glacial acetic acid, and biofilm biomass was quantitated by measuring the OD_560_. Values are from three biological replicates (mean ± SEM), and statistical values were determined by two-way ANOVA. ns, *P* > 0.05; *, *P* < 0.05; **, *P* < 0.01; ***, *P* < 0.001. (C and D) qRT-PCR of *psmα* (C) and *psmβ* (D) in biofilms. Static biofilms were grown in 12-well plates for 12 h at 37°C. The cDNA samples used were prepared from RNA isolated from cells grown in biofilms. Values are from three biological replicates (mean ± SEM). Statistical values were determined by one-way ANOVA. ***, *P* < 0.001. (E) Biofilm formation by NCTC8325 WT, Δ*α/β*, Δ*mgrA*, and Δ*mgrA/α/β* strains. Static biofilms were grown in 96-well plates at 37°C. At the indicated time points, biofilms were washed, stained with 0.5% crystal violet, and visualized with a camera. (F) Biofilm quantitation. Stained biofilms (E) were solubilized with 30% glacial acetic acid, and biofilm biomass was quantitated by measuring the OD_560_. Values are from three biological replicates (mean ± SEM), and statistical values were determined by two-way ANOVA. ns, *P* > 0.05; *, *P* < 0.05; **, *P* < 0.01; ***, *P* < 0.001. NCTC8325-pli50, NCTC8325 WT strain carrying plasmid pli50; Δ*mgrA*-pli50, *mgrA* mutant strain carrying plasmid pli50; Δ*mgrA*-pli*mgrA*, *mgrA* mutant strain carrying plasmid pli*mgrA*; NCTC8325 WT, NCTC8325 WT strain; Δ*α/β*, *psmα psmβ* double mutant strain; Δ*mgrA*, *mgrA* mutant strain; Δ*mgrA/α/β*, *mgrA psmα psmβ* triple mutant strain.

Previous studies showed that PSMs are involved in biofilm formation and detachment (21, 32), and MgrA represses biofilm formation in S. aureus (13). To demonstrate that MgrA can regulate biofilm development by repressing the expression of *psm* operons in S. aureus, we detected the biofilm formation in the WT, *mgrA* mutant, and *mgrA*-complemented strains and examined the transcriptional levels of *psm* genes in biofilms. Biofilm formation was significantly increased in the *mgrA* mutant strain, compared with the WT strain, after 12 h and 24 h of incubation, but biofilms of the *mgrA* mutant strain were much weaker than those of the WT strain after 36 h, 48 h, 60 h, and 72 h of incubation, and these changes could be reversed by introducing pli*mgrA* into the *mgrA* mutant strain (Fig. 4A). Similar results were observed when the stained biofilms were solubilized with 30% glacial acetic acid and quantitated by reading the optical density at 560 nm (OD_560_) (Fig. 4B). These results suggested that MgrA repressed biofilm formation at the early biofilm development stage (before 24 h of incubation) and repressed biofilm disassembly at the late biofilm development stage (after 36 h of incubation).

To determine whether MgrA could regulate the expression of *psm* operons in biofilms, the bacterial cells in biofilms were collected after 12 h of incubation and RNAs were extracted to quantitate the transcriptional levels of *psm* genes. The transcriptional levels of *psmα* and *psmβ* were significantly increased in the *mgrA* mutant strain, compared with the WT strain, and the changes could be reversed by introducing pli*mgrA* into the *mgrA* mutant strain (Fig. 4C and D). These findings indicated that MgrA could repress the expression of *psm* operons in cultures and biofilms. Thus, we speculated that MgrA could modulate biofilm formation by repressing the production of PSMs in S. aureus.

To test our speculation, we constructed the isogenic *psm* deletion strains and detected biofilm formation (Fig. 4E and F). Biofilm formation was significantly increased in the *psmα psmβ* double mutant strain, compared with the WT strain, which was consistent with a previous report (21). However, biofilm formation was decreased in the *mgrA psmα psmβ* triple mutant strain, compared with the *mgrA* mutant strain, after 18 h of incubation (Fig. 4E and F). These data suggested that the overexpression of *psm* genes could promote biofilm formation at the early biofilm development stage in the *mgrA* mutant strain. After 36 h of incubation, more biofilm was detected in the *mgrA psmα psmβ* triple mutant strain, compared with the *mgrA* mutant strain (Fig. 4E and F), suggesting that the overexpression of *psm* genes promoted biofilm disassembly quickly at the late biofilm development stage in the *mgrA* mutant stain. After 72 h of incubation, less biofilm was detected and no difference between the *mgrA* mutant and *mgrA psmα psmβ* triple mutant strains was observed, suggesting that biofilms detached totally in the *mgrA* mutant and *mgrA psmα psmβ* triple mutant strains (Fig. 4E and F). Collectively, these results indicated that MgrA could modulate biofilm development by repressing the expression of *psm* operons in S. aureus.

### MgrA modulates biofilm structuring at the early biofilm development stage by repressing the expression of *psm* operons.

**FIG 5:**
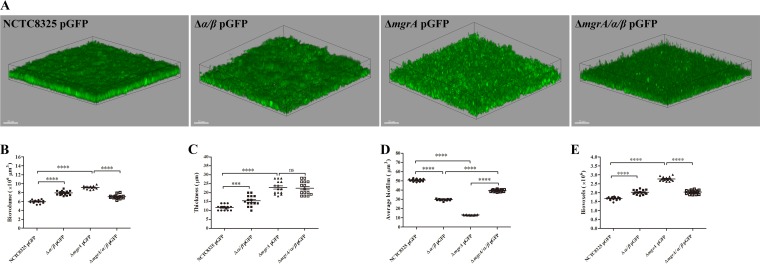
Static biofilm formation by S. aureus. Static biofilms were grown in chambered cover glass plates at 37°C for 12 h. Z-stacks were obtained with CLSM at 2-μm intervals, reconstructed with Zeiss ZEN 2012 software, and analyzed with Imaris 7.0 software. All confocal parameters were set with the settings for NCTC8325 pGFP biofilm as standard settings for comparing the biofilms produced by Δ*mgrA* pGFP, Δ*α/β* pGFP, and Δ*mgrA/α/β* pGFP strains. (A) Biofilm images of S. aureus strains. Biofilm images were obtained in at least 10 randomly chosen fields of the same biofilm. Extensions and scales are the same in all images (total extension on the *x* axis, 170 μm; total extension on the *y* axis, 170 μm). (B to E) Biofilm quantitation. Biofilm parameters were measured at 12 h in at least 15 randomly chosen biofilm CLSM images of the same extension. Horizontal bars depict the means. Statistical analysis was performed with *t* tests. ns, *P* > 0.05; ***, *P* < 0.001; ****, *P* < 0.0001. NCTC8325 pGFP, NCTC8325 WT strain carrying plasmid pGFP; Δ*mgrA* pGFP, *mgrA* mutant strain carrying plasmid pGFP; Δ*α/β* pGFP, *psmα psmβ* double mutant strain carrying plasmid pGFP; Δ*mgrA/α/β* pGFP, *mgrA psmα psmβ* triple mutant strain carrying plasmid pGFP.

Previous studies proved that PSMs are involved in biofilm structuring in the premier biofilm-forming pathogen S. aureus (21). To determine the changes in biofilm structuring in the *mgrA* mutant strain, we constructed a plasmid containing a green fluorescent protein (GFP)-coding gene, transformed it into the WT, *psmα psmβ* double mutant, *mgrA* mutant, and *mgrA psmα psmβ* triple mutant strains, and assessed biofilm samples with confocal laser scanning microscopy (CLSM) after 12 h of incubation (Fig. 5A). In static biofilms, total biofilm volume and mean thickness values were significantly greater in these mutants, compared with the WT strain, after 12 h of incubation (Fig. 5B and C), suggesting that both MgrA and PSMs can repress biofilm formation in the WT strain. Additionally, total biofilm volume was decreased in the *mgrA psmα psmβ* triple mutant strain, compared with the *mgrA* mutant strain, after 12 h of incubation (Fig. 5B), suggesting that PSMs can promote biofilm formation in the *mgrA* mutant strain. Furthermore, average biofilm volume values (indicating the degree of channel formation) were higher and total biofilm voxel values were lower in the *mgrA psmα psmβ* triple mutant strain, compared with the *mgrA* mutant strain (Fig. 5D and E), indicating that the *mgrA* mutant strain formed less compact and rougher biofilms on the surface and had more prominent biofilm channels. These results suggested that PSMs promoted biofilm structuring under static conditions in the *mgrA* mutant strain. Collectively, these findings indicated that MgrA can modulate biofilm structuring by repressing the production of PSMs in S. aureus.

### The *mgrA* mutant strain exhibits increased dynamic biofilm formation and detachment.

**FIG 6:**
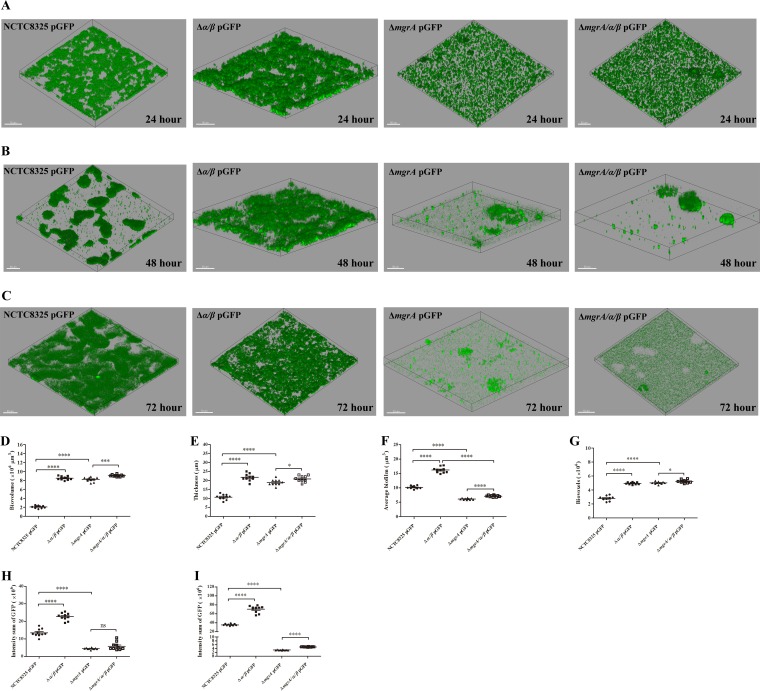
Dynamic S. aureus biofilm formation and detachment. (A to C) Biofilm images. Dynamic biofilms were grown at 37°C in the flow cell system. At the indicated time points, z-stacks were obtained with CLSM at 1-μm intervals, reconstructed with Zeiss ZEN 2012 software, and analyzed with Imaris 7.0 software. All confocal parameters were set with the settings for NCTC8325 pGFP biofilm as standard settings for comparing the biofilms produced by Δ*mgrA* pGFP, Δ*α/β* pGFP, and Δ*mgrA/α/β* pGFP strains. Biofilm images were obtained at 24 h (A), 48 h (B), and 72 h (C) in at least 10 randomly chosen fields of the same biofilm. Extensions and scales are the same in all images (total extension on the *x* axis, 210 μm; total extension on the *y* axis, 210 μm). (D to G) Biofilm quantitation. Biofilm parameters were measured at 24 h of incubation in at least 10 randomly chosen biofilm CLSM images of the same extension. (H and I) Intensity sum of GFP for the 48-h biofilms (H) and the 72-h biofilms (I). Horizontal bars depict the means. Statistical analysis was performed with *t* tests. ns, *P* > 0.05; *, *P* < 0.05; ***, *P* < 0.001; ****, *P* < 0.0001. NCTC8325 pGFP, NCTC8325 WT strain carrying plasmid pGFP; Δ*mgrA* pGFP, *mgrA* mutant strain carrying plasmid pGFP; Δ*α/β* pGFP, *psmα psmβ* double mutant strain carrying plasmid pGFP; Δ*mgrA/α/β* pGFP, *mgrA psmα psmβ* triple mutant strain carrying plasmid pGFP.

Biofilm formation is commonly analyzed under static or dynamic (flow cell-grown) conditions, and these conditions may be strongly divergent. Therefore, we measured biofilm development under dynamic conditions using the flow cell system. First, we compared the biofilm formation of the WT, *psmα psmβ* double mutant, *mgrA* mutant, and *mgrA psmα psmβ* triple mutant strains at the early biofilm development stage. Biofilm formation was increased in all of these mutant strains, compared with the WT strain, after 24 h of incubation (Fig. 6A, D, and E). More biofilm was detected in the *psmα psmβ* double mutant strain than in the WT strain, and the *mgrA* mutant strain formed a little less biofilm than did the *mgrA psmα psmβ* triple mutant strain (Fig. 6D). These data indicated that MgrA and PSMs could repress biofilm formation in the WT strain and PSMs could slightly repress biofilm formation under dynamic conditions at the early biofilm development stage in the *mgrA* mutant strain. To further investigate what changes would occur as the incubation time was extended, we assessed biofilm development successively and found that the *mgrA* mutant and *mgrA psmα psmβ* triple mutant strains exhibited fast biofilm detachment, while the WT and *psmα psmβ* double mutant strains kept stable biofilm growth (Fig. 6B and C). Moreover, compared with the *mgrA psmα psmβ* triple mutant strain, the biofilms of the *mgrA* mutant strain disassembled more quickly and had less biofilm volume after 48 h (Fig. 6B and H) and 72 h (Fig. 6C and I) of incubation. These results suggested that MgrA repressed biofilm detachment by controlling the production of PSMs in biofilms under dynamic conditions. We also measured other biofilm parameters of 24-h biofilms (Fig. 6F and G). Average biofilm volume values and total biofilm voxel values were higher in the *psmα psmβ* double mutant strain, compared with the WT strain, indicating that the *psmα psmβ* double mutant strain formed more compact biofilms on the surface and had more prominent biofilm channels. Average biofilm volume values were lower and total biofilm voxel values were higher in the *mgrA* mutant strain, compared with the WT strain, indicating that the *mgrA* mutant strain formed less compact biofilms on the surface and had more prominent biofilm channels. Moreover, average biofilm volume values were a little higher in the *mgrA psmα psmβ* triple mutant strain than in the *mgrA* mutant strain, indicating that the *mgrA* mutant strain formed slightly less compact biofilms on the surface. These results allowed us to conclude that PSMs promoted biofilm structuring, under dynamic conditions, at the early biofilm development stage in the WT and *mgrA* mutant strains. Collectively, these results suggested that MgrA repressed biofilm formation and structuring at the early biofilm development stage and weakened biofilm detachment at the late biofilm development stage by modulating the production of PSMs in biofilms.

## DISCUSSION

PSMs are amphipathic and surfactant-like peptides that play multiple roles in biofilm formation and virulence in S. aureus. Despite the multiple functions of PSMs in S. aureus, the regulatory mechanism controlling the expression of *psm* operons has not been thoroughly elucidated. AgrA, a regulator of the *agr* quorum-sensing system, is a positive regulator of *psm* operons and strictly regulates *psm* expression by binding directly to the promoter regions of *psm* operons (20). No other regulators that could directly modulate *psm* expression in S. aureus have been reported. In this study, we have demonstrated that MgrA can negatively regulate the expression of *psm* genes by binding directly to the promoter regions of *psm* operons. Thus, the functions of MgrA and AgrA in the regulation of *psm* genes in S. aureus seem to be opposite. Since no PSMs are produced in *agr*-negative strains (20), we tried to restore the production of PSMs by inactivating MgrA in *agr*-negative strains. However, the deletion of *mgrA* could not restore the production of PSMs in *agr*-negative strains (see Fig. S2 in the supplemental material). Previous studies suggested that MgrA and the *agr* system can regulate each other in S. aureus. For instance, MgrA can regulate the expression of the *agr* system and virulence genes (29), and RNAIII, which is encoded by the *agr* system, can modulate the production of MgrA (33). It seems that AgrA and MgrA play roles in several common regulatory pathways and AgrA is a positive and indispensable regulator of PSMs in S. aureus. To our surprise, other regulators, such as AgrA, were not identified in our pulldown assay. However, we could not exclude the possibility that other transcriptional regulators are involved in the modulation of *psm* expression.

MgrA regulates biofilm- and virulence-associated genes by binding directly to the promoter regions of target genes in S. aureus (16, 17, 19). MgrA activates *sarX* transcription by binding upstream of the −35 box region on the promoter of *sarX* (16), represses *sarV* expression by binding upstream of the −35 box region on the promoter of *sarV* (17), and represses the expression of surface proteins by binding to the −10 and −35 promoter regions of those genes (19). In this study, we have demonstrated that MgrA can repress *psm* expression by binding directly to the −10 and −35 promoter regions of *psm* operons, which is consistent with previous studies. However, the microarray analysis by Crosby et al. failed to identify *psm* operons as members of the MgrA regulon (19). It has been reported that the transcription of *psm* operons is strictly controlled by AgrA (20). As we know, the expression of *psm* genes is initiated when the *agr* quorum-sensing system is activated at a certain threshold level of bacterial cell density in the postexponential phase (24). Crosby et al. performed RNA sequencing to identify genes regulated by *mgrA* in S. aureus strain LAC (19). On the basis of the RNA sequencing data, they failed to identify *psm* operons as members of the MgrA regulon, most likely due to the threshold for microarray data. In their study, S. aureus strain LAC and the isogeneic *mgrA* mutant were grown in rich medium to an OD_600_ of 1.5, a point at which the bacterial cell density is not enough to activate the *agr* quorum-sensing system, leading to a low level of *psm* operon transcription and the failure of RNA sequencing to identify *psm* operons as members of the MgrA regulon.

MgrA modulates biofilm formation by controlling surface protein expression, protease production, and eDNA release in S. aureus (13, 19). Our data indicated that the *mgrA* mutant developed increased biofilm formation at the early biofilm development stage (before 24 h of incubation). This result is consistent with the report by Trotonda et al. (13). However, our findings showed decreased biofilm formation at the late biofilm development stage in the *mgrA* mutant strain (Fig. 4 and 6). These data are not completely in agreement with previous studies, which showed that MgrA repressed biofilm formation in S. aureus (13, 15, 19). This difference can be explained by the finding that MgrA can repress the expression of *psm* operons in our strain. On one hand, the development of biofilms contains three stages, including attachment, maturation, and detachment (9). Thus, the biofilms begin to disassemble at the late biofilm development stage (after 36 h of incubation). On the other hand, PSMs are surfactant-like peptides and can promote biofilm disassembly in S. aureus (21). At the late biofilm development stage, large amounts of PSMs were produced, which can promote biofilm disassembly in the *mgrA* mutant strain. As a result, the *mgrA* mutant strain had less biofilm than did the WT strain at the late biofilm development stage. In fact, our findings are not totally opposed to and can be taken as a supplement to previous studies.

Although PSMs are associated with biofilm structuring, biofilms are also significantly reduced by PSMs due to the surfactant-like properties of PSMs (21). However, the *mgrA* mutant strain developed enhanced biofilm formation along with increased expression of *psm* genes at the early biofilm development stage. We considered that other biofilm-associated factors regulated by MgrA, such as the increased eDNA release in the *mgrA* mutant strain, might contribute to the enhancement of biofilm formation (Fig. S3). Indeed, autolysis of the *mgrA* mutants is increased, and the release of eDNA is increased in biofilms of the *mgrA* mutant strain (13, 29, 34). Several studies have shown that eDNA is a component of biofilms and can promote biofilm formation in S. aureus (35–38). Moreover, MgrA can regulate the production of nucleases (39), proteases (13), and surface proteins (19) to modulate biofilm formation.

Additionally, it has been reported that PSMs are involved in the pathogenesis of S. aureus (8, 24) and that MgrA can modulate the expression of virulence-associated genes in S. aureus (16, 19, 29). In this study, we found that the ability to lyse blood cells and epithelial cells was decreased in the *mgrA psmα psmβ* triple mutant strain in comparison with the *mgrA* mutant strain and was increased in the *mgrA psmα psmβ* triple mutant strain in comparison with the *psmα psmβ* double mutant strain (Fig. S4). These results suggested that MgrA may modulate the pathogenesis of S. aureus by repressing the production of PSMs *in vivo*.

In conclusion, our findings have revealed that MgrA is a negative regulator of *psm* genes and represses the production of PSMs by binding directly to the promoter regions of the *psm* operons. Our findings provide novel insights into the regulatory mechanisms of *psm* gene expression, biofilm development, and S. aureus pathogenesis.

## MATERIALS AND METHODS

### Bacterial strains, plasmids, and growth conditions.

The bacterial strains and plasmids used in this study are listed in Table 3. S. aureus cells were grown at 37°C, with aeration, in tryptic soy broth (TSB; BD) supplemented with antibiotics when necessary. Luria-Bertani (LB) medium (Oxoid) was used for cultivation of Escherichia coli. Antibiotics were used at the following concentrations: for S. aureus, chloramphenicol (Cm) at 15 μg/ml; for E. coli, ampicillin at 150 μg/ml and kanamycin at 50 μg/ml.

**TABLE 3:** Strains and plasmids used in this study

Strain or plasmid	Description^*a*^	Source or reference
Strains		
Escherichia coli		
Trans-T1	Cloning strain	TransGen
BL21(DE3)	Expression strain	TransGen
Staphylococcus aureus		
NCTC8325 WT	NCTC8325 WT strain	NARSA^*b*^
RN4220	8325-4; restriction modification deficient	NARSA
Δ*mgrA*	NCTC8325 *mgrA* mutant strain	This study
Δα*/β*	NCTC8325 *psmα psmβ* double mutant strain	This study
Δ*mgrA/α/β*	NCTC8325 *mgrA psmα psmβ* triple mutant strain	This study
Δ*agr*	NCTC8325 *agr* mutant strain	This study
Δ*agr*/*mgrA*	NCTC8325 *agr mgrA* double mutant strain	This study
N315 WT	N315 (*agr*-negative) WT strain	NARSA
N315Δ*mgrA*	N315 *mgrA* mutant strain	This study
NCTC8325-pli50	NCTC8325 carrying plasmid pli50	This study
Δ*mgrA*-pli50	Δ*mgrA* carrying plasmid pli50	This study
Δ*mgrA*-pli*mgrA*	Δ*mgrA* carrying plasmid pli*mgrA*	This study
Δα*/β*-pli50	Δα*/β* carrying plasmid pli50	This study
Δ*mgrA/α/β*-pli50	Δ*mgrA/α/β* carrying plasmid pli50	This study
NCTC8325 pGFP	NCTC8325 carrying plasmid pGFP	This study
Δα*/β* pGFP	Δα*/β* carrying plasmid pGFP	This study
Δ*mgrA* pGFP	Δ*mgrA* carrying plasmid pGFP	This study
Δ*mgrA/α/β* pGFP	Δ*mgrA/α/β* carrying plasmid pGFP	This study
Plasmids		
pBTs	E. coli-S. aureus shuttle vector; temperature sensitive, Amp^r^, Cm^r^	43
pBTs-*mgrA*	pBTs plasmid containing mutant allele for *mgrA* deletion; Amp^r^, Cm^r^	This study
pBTs-*psmα*	pBTs plasmid containing mutant allele for *psmα* deletion; Amp^r^, Cm^r^	This study
pBTs-*psmβ*	pBTs plasmid containing mutant allele for *psmβ* deletion; Amp^r^, Cm^r^	This study
pli50	E. coli-S. aureus shuttle vector; Amp^r^, Cm^r^	Addgene
pli*mgrA*	Complete *mgrA* gene under control of native promoter in pli50; Amp^r^, Cm^r^	This study
pGFP	*gfp* expression with promoter of S10 ribosomal gene; Amp^r^, Cm^r^	49

a Amp^r^, ampicillin resistant; Cm^r^, chloramphenicol resistant.

b NARSA, Network on Antimicrobial Resistance in Staphylococcus aureus.

### Genetic manipulation in E. coli and S. aureus.

Construction of recombinant plasmids was performed in E. coli Trans-T1 with standard molecular biology and recombinant DNA techniques. Genomic DNA of S. aureus was prepared by using a standard protocol for Gram-positive bacteria (40). Plasmid DNA was extracted with a plasmid purification kit (Promega), according to the manufacturer's instructions. All plasmids transformed into the indicated S. aureus strains were introduced first into S. aureus RN4220 for modification by electroporation, as described previously (41).

To construct the isogenic *mgrA* deletion strain, two fragments flanking upstream and downstream of *mgrA* were amplified from S. aureus NCTC8325 genomic DNA with the primer pairs *mgrA*-up-F/*mgrA*-up-R and *mgrA*-down-F/*mgrA*-down-R (Table 4). The two PCR products had a 20-base complementary region to facilitate ligation followed by the seamless ligation cloning extract (SLiCE) method, as described previously (42). Outside primers *mgrA*-up-F and *mgrA*-down-R were then used to amplify a single fragment from the ligated products, and the fusion product was purified, digested with KpnI and SalI, and cloned into the shuttle vector pBTs to generate pBTs-*mgrA* (Table 3). The plasmid pBTs-*mgrA* was transformed first into RN4220 at 30°C on tryptic soy agar (TSA) containing 15 μg/ml Cm (TSA Cm plates) for modification and then into the WT and *agr* mutant strains by electroporation. Allelic exchange in the absence of the selection marker was performed as described previously (43). Briefly, individual colonies were streaked on TSA Cm plates and incubated at 30°C. Single colonies were grown in TSB at 42°C and diluted 1:200 in fresh medium for 3 successive days before being diluted to 10^−3^ and plated on TSA Cm plates to select for integration into the chromosome. Single colonies were grown in TSB at 30°C and diluted 1:200 in fresh medium for 2 successive days before being diluted to 10^−6^ and plated on TSA plates containing 0.1 μg/ml anhydrotetracycline to select for loss of the plasmid. Colonies were screened for resistance to Cm, and Cm-sensitive colonies were screened for deletion of *mgrA* by PCR. The *mgrA* mutants were verified by PCR and sequencing. A similar strategy was used to construct S. aureus agr and *psm* deletion mutant strains. All primers used in this study are listed in Table 4.

**TABLE 4:** Sequences of primers used in this study

Primer name	Sequence (5′ to 3′)^*a*^	Modification
P*psmα*-F	CCGGAATTCTCTGTTCAATTCATCTTCATA	
P*psmα*-biotin-R	CGCGGATCCCCGCCAGCGATGATACCCATTAAG	5′-Biotin
P*rbf*-F	CCGGAATTCCAGGTGTACTTGCCTTTCTA	
P*rbf*-biotin-R	CGCGGATCCCCCAAGCATGATTTTGCCATAAC	5′-Biotin
P*psmα*-F	CCGGAATTCTCTGTTCAATTCATCTTCATA	
P*psmα*-R	CGCGGATCCCCGCCAGCGATGATACCCATTAAG	
P*psmβ*-F	ACTTAAATACGAATTCAGGCAACT	
P*psmβ*-R	AACCTTCCATTGAAAACACTCC	
P*psmβ*-biotin-R	AACCTTCCATTGAAAACACTCC	5′-Biotin
*pta*-F	AAAGCGCCAGGTGCTAAATT	
*pta*-R	CTGGACCAACTGCATCATAT	
P*psmα*-FAM-F	TCTGTTCAATTCATCTTCATA	5′-6-FAM
P*psmβ*-FAM-F	ACTTAAATACGAATTCAGGCAACT	5′-6-FAM
*mgrA*-up-F	GCGggtaccATGTCACTTAGTTTCAAC	
*mgrA*-up-R	TTACCTAATAAGCGATTAAGTGCTGTTCTTTTAAATTATG	
*mgrA*-down-F	ACTTAATCGCTTATTAGGTAA	
*mgrA*-down-R	GCGgtcgacCAGGGTTATATCAATTAGATAG	
*psmα*-up-F	GCGGAATTCGAGCTCggtaccAATGTAATACCCCAGCAGAGTGCC	
*psmα*-up-R	GGACGGGAAATACTGCCTAACTGT	
*psmα*-down-F	ACAGTTAGGCAGTATTTCCCGTCCTCTCAGGCCACTATACCAATAGGG	
*psmα*-down-R	CTTGCATGCCTGCAGGTCGACCCAGAATATGGCGATCGTCA	
*psmβ*-up-F	GCGGAATTCGAGCTCggtaccCCAGAATATGGCGATCGTCA	
*psmβ*-up-R	CAATTAGTTTGTTTATCCGCACA	
*psmβ*-down-F	TGTGCGGATAAACAAACTAATTGCAATTAGTTTGTTTATCCGCACA	
*psmβ*-down-R	CTTGCATGCCTGCAGGTCGACGTGCTGTCTTTCATCCTCACCA	
*mgrA*-c-F	GCGggatccTCATCATTTTTTAATAAT	
*mgrA*-c-R	GCGgtcgacCAATTACTAGCTAATCAAGG	
*mgrA*-RT-F	GACAAGTTAATCGCTACTAC	
*mgrA*-RT-R	GAGTGCTAATTCAGTTACG	
*psmα*-RT-F	GTATCATCGCTGGCATCA	
*psmα*-RT-R	AAGACCTCCTTTGTTTGTTATG	
*psmβ*-RT-F	TGGACTAGCAGAAGCAATC	
*psmβ*-RT-R	TAGTAAACCCACACCGTTAG	
*hld*-RT-F	GTGAATTTGTTCACTGTGTCGA	
*hld*-RT-R	GGAGTGATTTCAATGGCACAAG	
*hu*-RT-F	AAAAAGAAGCTGGTTCAGCAGTAG	
*hu*-RT-R	TTTACGTGCAGCACGTTCAC	

a The sequences in lowercase letters refer to restriction endonuclease recognition sites.

Complementation plasmids were created as follows. The complete *mgrA* gene with its native promoter was amplified by PCR with primers *mgrA*-c-F and *mgrA*-c-R (Table 4). The resulting product was digested with BamHI and SalI and ligated with pli50, which had been digested with the same enzymes, to generate pli*mgrA*. The complementing plasmids were transformed into S. aureus RN4220 and then into the *mgrA* mutant strain. The WT and *mgrA* mutant strains were also transformed with the pli50 plasmid, which was used as the control.

### DNA pulldown assay.

The DNA pulldown assay was performed as described previously (40, 44), with minor modifications. Briefly, the biotin-labeled DNA fragment containing the promoter region was amplified from S. aureus NCTC8325 genomic DNA using primers P*psmα*-F and P*psmα*-biotin-R (Table 4). The control DNA fragment of the *rbf* promoter sequence was amplified with primers P*rbf*-F and P*rbf*-biotin-R (45). S. aureus cultures at different growth phases were collected and were resuspended in lysis buffer (10 mM HEPES [pH 7.0], 200 mM NaCl, 1% Triton X-100, 10 mM MgCl_2_, 1 mM dithiothreitol) containing protease inhibitor cocktail (Sangon), 1 U/ml DNase I (Promega), and 40 U/ml lysostaphin (AMBI). To lyse the cells completely, the suspension was incubated at 37°C with shaking until the cells were thoroughly lysed. The lysate was centrifuged at 12,000 × *g* for 30 min at 4°C to remove insoluble debris, and the supernatants were then collected. The total protein was stored temporarily at 4°C for later use. At the same time, streptavidin-MagneSphere paramagnetic particles (SA-PMPs) (Promega) were rinsed twice with 0.5× standard saline citrate (SSC) (1× SSC is 0.15 M NaCl plus 0.015 M sodium citrate) and then rinsed twice with lysis buffer. The DNA sample (200 μl; 200 to 400 ng/μl) was incubated with the prepared SA-PMPs at 25°C for 1 h on a rotating shaker; total protein was then added, and the mixture was incubated at 4°C for 1.5 h on a rotating shaker. The SA-PMPs was then washed three times with fresh lysis buffer. The proteins captured were incubated at 95°C for 10 min, separated by SDS-PAGE, and then stained with silver or brilliant blue R-250. The gel bands were excised and digested with trypsin (0.6 mg), and the tryptic peptides were subjected to LC-MS/MS analysis with a linear trap quadrupole (LTQ) mass spectrometer (ProteomeX-LTQ; Thermo Fisher Scientific). Sequence and peptide fingerprint data were analyzed using the NCBI database.

### MgrA expression and purification.

The 444-bp DNA fragment containing the complete *mgrA* gene (SAOUHSC_00694 of S. aureus NCTC8325) was amplified from S. aureus NCTC8325 genomic DNA using primers containing flanking restriction sites (NdeI and XhoI) to facilitate in-frame cloning into the expression vector pET28a(+) to obtain pET*mgrA*. The recombinant plasmid containing the *mgrA* coding region was confirmed by restriction digestion analysis and DNA sequencing and was subsequently transformed into E. coli BL21(DE3). The expression and purification of the recombinant His6-MgrA protein were performed as described previously (17). The purity of the purified His6-MgrA fusion protein was determined by SDS-PAGE, with brilliant blue R-250 staining. The concentration of the purified MgrA protein was determined with the Bradford protein assay (Bio-Rad, Hercules, CA), using bovine serum albumin as the standard.

### EMSA.

The 5′-biotin-labeled DNA fragments containing the promoter region were amplified from S. aureus NCTC8325 genomic DNA and incubated at 25°C for 1 h with various amounts of MgrA in 8 μl of incubation buffer (50 mM Tris-HCl [pH 8.0], 200 mM NaCl). After incubation, 2 μl of gel loading buffer was added to the mixtures, and then the mixtures were electrophoresed in a 5% native polyacrylamide gel in 1× Tris-borate-EDTA buffer. The band shifts were detected and analyzed with the chemiluminescent nucleic acid detection module (Pierce), according to the manufacturer's instructions. Images were obtained using an ImageQuant LAS 4000 mini imager (GE, Piscataway, NJ). Unlabeled promoter fragments in 100-fold (or 50-fold) excess were added as specific competitors. An unlabeled DNA fragment of the housekeeping gene *pta* in 100-fold (or 50-fold) excess was added as a nonspecific competitor.

### DNase I footprinting assay.

Footprinting assays with the *psmα* promoter region and DNase I were performed as described previously (46). The forward primer was synthesized and 5′ labeled with 6-FAM. The labeled DNA fragment was amplified from S. aureus NCTC8325 genomic DNA by PCR. The binding reactions were carried out for 1 h at 25°C in a 100-μl reaction volume containing 20 mM Tris-HCl (pH 8.0), 100 mM NaCl, 5 mM MgCl_2_, 1 mM CaCl_2_, 2 mM dithiothreitol, 1 pmol 5′-6-FAM-labeled template DNA, and various amounts of purified MgrA. DNase I (0.1 U in 100 μl; Promega) was added to the reaction mixture, the mixture was incubated for 1 min at 37°C, and then DNA was extracted with phenol-chloroform, precipitated with 95% ethanol, washed with 75% ethanol, dried, and dissolved in double-distilled water. DNA samples were detected by short tandem repeat sequencing. The protected region of MgrA was derived by comparing the sequencing results with versus without MgrA, using Peak Scanner software (v1.0; Applied Biosystems).

### Total RNA isolation, cDNA generation, and qRT-PCR.

Total RNA was prepared with a TRIzol isolation kit (TaKaRa) and a reciprocating shaker, as described previously (40). Briefly, overnight cultures of S. aureus were diluted 1:200 in fresh TSB containing 15 μg/ml Cm, grown at 37°C with shaking (220 rpm), and then grown to the indicated OD_600_ before being collected. The collected cells were processed with 1 ml of RNAiso Plus reagent (TaKaRa) in combination with 0.1-mm-diameter silica beads in a FastPrep-24 automated system (MP Biomedicals, Solon, OH), and the residual DNA was removed with RNase-free DNase I (TaKaRa). The concentration of total RNA was determined by measuring the absorbance at 260 nm, and the concentration of total RNA was adjusted to 200 ng/μl. For reverse transcription, cDNA templates were synthesized from 200 ng of total RNA with the PrimeScript first-strand cDNA synthesis kit (TaKaRa). qRT-PCR was performed with SYBR Premix Ex Taq reagent (TaKaRa), using the StepOne real-time PCR system (Applied Biosystems) and following the MIQE guidelines. The housekeeping gene *hu* was used as an endogenous control, and the quantity of cDNA measured through qRT-PCR was normalized to the abundance of *hu* cDNA, as described previously (47). The qRT-PCR specificity was confirmed using a melting curve of the products. To assess DNA contamination, each RNA sample was subjected to qRT-PCR using SYBR Premix Ex Taq reagent (TaKaRa). To determine the reaction efficiency of qRT-PCR, a series of diluted cDNA samples of the WT strain were subjected to qRT-PCR using SYBR Premix Ex Taq reagent (TaKaRa). Relative expression levels were determined by the comparative threshold cycle (ΔΔ*C_T_*) method. qRT-PCR was repeated three times in triplicate parallel experiments.

### Biofilm formation and analysis.

Biofilm formation under static conditions was determined by microtiter plate assays, based on the method described previously (13). Briefly, overnight cultures were diluted 1:200 in fresh TSB and inoculated (200 μl in each well) in flat-bottom 96-well polystyrene plates (Costar), in triplicate. The plates were incubated at 37°C for different times, and the wells were washed gently three times with water (to remove nonadherent cells), stained with 0.5% crystal violet for 5 min, and then again washed three times with water. To visualize biofilms, photographs of the inverted plate were taken with a camera. To quantitate the biofilms, the crystal violet stain was solubilized with 30% glacial acetic acid for 15 min, and the relative biofilm formation was determined by reading OD_560_ values using an enzyme-linked immunosorbent assay reader (Bio-Tek).

For CLSM, static biofilms were grown in chambered cover glass plates, as described previously (21), and analyzed with a Zeiss LSM 700 confocal microscope after gentle washing. Dynamic biofilms were grown in a flow cell system (Stovall Life Science, Greensboro, NC), which was assembled and prepared according to the manufacturer's instructions. Dynamic biofilm formation and CLSM were performed as described previously (48). Overnight cultures of different strains were adjusted to an OD_600_ of 5 and diluted 1:200 in fresh 0.2% TSB containing 15 μg/ml Cm and 0.2% glucose. The flow cells were inoculated with 5 ml of the culture dilutions and incubated at 37°C for 1 h. After this incubation period, the culture medium was continually perfused over the flow cell system, using a high-precision tubing pump with a planetary drive (ISMATEC, Switzerland), at a rate of 0.5 ml/min. Biofilms of different strains, which were transformed with the GFP plasmid for fluorescence detection, were cultivated at 37°C in three individual channels in 0.2% TSB containing 15 μg/ml Cm and 0.2% glucose. CLSM was performed with a Zeiss LSM710 system (Carl Zeiss, Jena, Germany) with a 20× apochromatic objective (numerical aperture of 0.8), fluorescence was excited with an argon laser at 488 nm, an emission band-pass filter of 515 ± 15 nm was used, and z-stacks were collected at 1-μm intervals. Confocal parameters set for detection of the WT biofilm were taken as the standard settings. Selected confocal images represented different areas of the biofilms and were selected from at least 10 different microscopic fields. Confocal images were acquired with the Zeiss ZEN 2010 software package (Carl Zeiss). Three-dimensional biofilm images were reconstructed with Zeiss ZEN 2012 software (Carl Zeiss) and analyzed with Imaris 7.0 software (Bitplane, Zurich, Switzerland).

## Supplementary Material

Supplemental material
