# Supplementary material for: MgrA Negatively Regulates Biofilm Formation and Detachment by Repressing the Expression of psm Operons in Staphylococcus aureus
Source: Appl Environ Microbiol. 2018 Aug 1;84(16):e01008-18. doi: 10.1128/AEM.01008-18 (PMC6070752; doi:10.1128/AEM.01008-18)
Supplement: Supplemental material [file supp_84_16_e01008-18__index.html]

Supplemental material 

# MgrA Negatively Regulates Biofilm Formation and Detachment by Repressing the Expression of *psm* Operons in Staphylococcus aureus

## Supplemental material

- Supplemental file 1 -

  List of non-DNA-binding proteins identified by LC-MS/MS (Table S1); detection of PSMs in the WT and the *mgrA* mutant strain (Fig. S1); the regulatory effects of MgrA on the expression of *psm* genes in the *agr*-negative strain (Fig. S2); eDNA levels in biofilms (Fig. S3); cell lysis ability of culture filtrates (Fig. S4).

  PDF, 502K
