# Supplementary material for: MgrA Negatively Regulates Biofilm Formation and Detachment by Repressing the Expression of psm Operons in Staphylococcus aureus
Source: Appl Environ Microbiol. 2018 Aug 1;84(16):e01008-18. doi: 10.1128/AEM.01008-18 (PMC6070752; doi:10.1128/AEM.01008-18)
Supplement: Supplemental material [file AEM.01008-18_zam016188670s1.pdf]

SUPPLEMENTAL MATERIALS

TABLE S1 List of non-DNA-binding proteins identified by LC-MS/MS

**Proteins**	**Genes**	**Molecular functions**	**MW (Da)**	**Score of abundance**
Transketolase	SAOUHSC _01337	Metal ion binding; Transketolase activity	68317.2	40.27
50S ribosomal protein	SAOUHSC _02509, *rplB*	RNA binding; rRNA binding; Structural constituent of ribosome; transferase activity.	30136.5	20.23
Pyruvate dehydrogenase complex, E1 component subunit beta	SAOUHSC _01041	Pyruvate dehydrogenase (acetyl-transferring) activity.	35224.2	20.19
Elastin-binding protein EbpS	SAOUHSC _01501, *ebpS*	Promotes binding of soluble elastin peptides and tropoelastin to *S.aureus* cells.	53190.2	10.22
Elongation factor P	SAOUHSC _01625, *efp*	Translation elongation factor activity.	20541.3	10.20
Hypothetical protein SAOUHSC_01969	SAOUHSC _01969	Uncharacterized protein.	13203.8	10.19
Ribose-phosphate pyrophosphokinase	SAOUHSC _00472, *prs*	ATP binding; kinase activity; Magnesium ion binding; Ribose phosphate diphosphokinase activity.	35261.6	10.19
ATP synthase subunit beta	SAOUHSC _02341, *atpD*	ATP binding; Proton-transporting ATP synthase activity, rotational mechanism.	51368.3	10.17
50S ribosomal protein L3	SAOUHSC _02512, *rplC*	rRNA binding; Structural constituent of ribosome.	23703.5	10.16
UPF0173 metal-dependent hydrolase	SAOUHSC _01815	Hydrolase activity.	25234.5	10.16
HMG-CoA synthase	SAOUHSC _02860	Hydroxymethylglutaryl-CoA synthase activity.	43178.5	10.16
Uridylate kinase	SAOUHSC _01235,	ATP binding; UMP kinase activity.	26128.4	10.16

	pyrH			
Aspartyl/glutamyl-tRNA(Asn/Gln) amidotransferase	SAOUHSC_02116, *gatB*	ATP binding; Glutaminyl-tRNA synthase.	53623.2	10.15
Hypothetical protein SAOUHSC_02699	SAOUHSC_02699	Uncharacterized protein, ionotropic glutamate receptor activity.	28886.0	10.15
30S ribosomal protein S2	SAOUHSC_01232, *rspB*	Structural constituent of ribosome.	29076.2	10.15
Acetyl-CoA acetyltransferase	SAOUHSC_00336	Acetyl-CoA C-acetyltransferase activity.	41810.3	10.15
50S ribosomal protein L4	SAOUHSC_02511, *rplD*	RNA binding; rRNA binding; Structural constituent of ribosome.	22451.0	10.15
Pyridoxal 5'-phosphate synthase subunit PdxS; Sho	SAOUHSC_00499, *pdxS*	Pyridoxal 5'-phosphate synthase (glutamine hydrolysing) activity.	31972.2	10.15
Glutamine synthetase	SAOUHSC_01287	ATP binding; glutamate-ammonia ligase activity.	50808.5	10.15
L-lactate dehydrogenase 1	SAOUHSC_00206, *ldh 1*	L-lactate dehydrogenase activity.	34562.0	10.14
NADP-dependent malic enzyme	SAOUHSC_01810	Malate dehydrogenase (decarboxylating) (NAD+) activity; Metal ion binding NAD binding.	44205.9	10.14
Peptide chain release factor 1	SAOUHSC_02359, *prfA*	Translation release factor activity, codon specific.	40325.3	10.14
Catalase	SAOUHSC_01327, *katA*	Catalase activity; Heme binding; Metal ion binding.	58343.3	10.13
Phosphomethylpyrimi-dine kinase	SAOUHSC_02330	ATP binding; Phosphomethylpyrimidine kinase activity	29837.9	10.13
Molybdenum ABC transporter permease	SAOUHSC_02547	Molybdate ion transmembrane transporter activity	24846.9	10.13

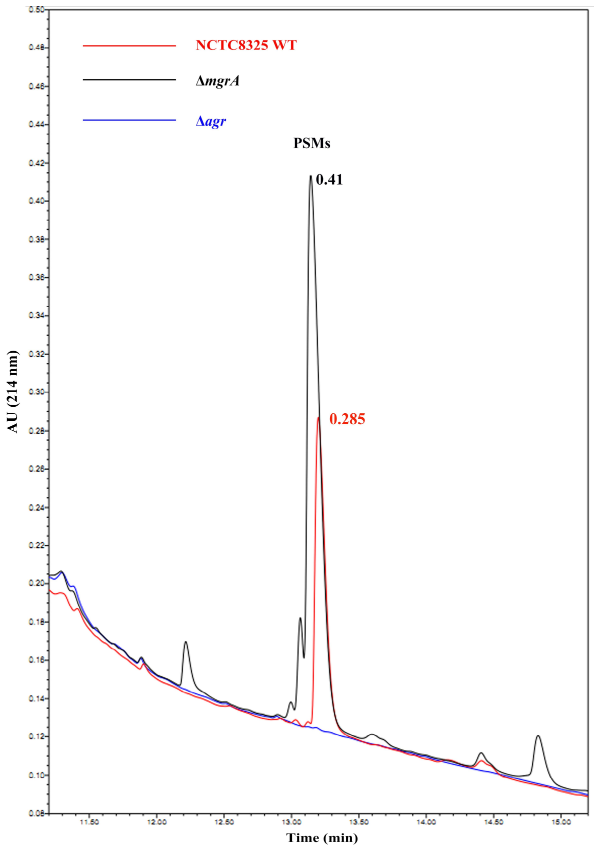

Figure S1. Detection of PSMs in the WT and the *mgrA* mutant strain. RP-UPLC of culture filtrates. NCTC8325 WT, Δ*mgrA*, and Δ*agr* were grown to stationary phase (10h) and collected. Culture filtrates were analyzed by RP-UPLC. The chromatography was performed using a column (ACQUITY UPLC BEH C18 1.7μm 2.1×50mm Colum) and a water/acetonitrile gradient in 0.1% trifluoroacetic acid from 0 to 100% acetonitrile at a flow rate of 0.5 ml/min. The UV signal at 214 nm is shown. The culture filtrate of the *agr* mutant strain was used as the negative control. Abbreviations: NCTC8325 WT, the NCTC8325 WT strain; Δ*mgrA*, the *mgrA* mutant strain; Δ*agr*, the *agr* mutant strain.

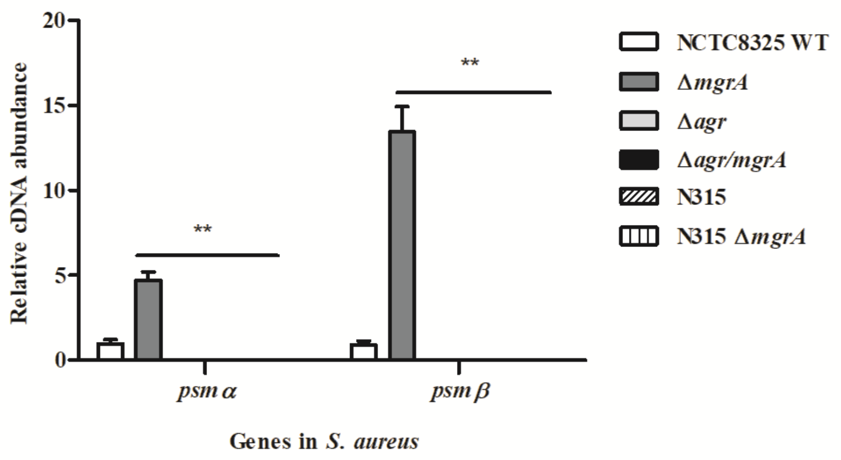

Figure S2. The regulatory effects of MgrA on the expression of *psm* genes in the *agr*-negative strain. Quantitative real-time PCR of *psm* genes in the NCTC8325 WT, Δ*mgrA*, Δ*agr*, Δ*agr/mgrA*, N315 WT, and N315 Δ*mgrA*. Bacteria were grown in cultures at 37℃ with shaking. cDNA samples used were prepared from RNA isolated from cells grown to stationary phase (10h). Probes were designed to align within partial regions of *psm* operons. Values are from three biological replicates, and statistical values were determined by the Student t test, **, $P<0.01$. Abbreviations: NCTC8325 WT, the NCTC8325 WT strain; Δ*mgrA*, the mgrA mutant strain; Δ*agr*/*mgrA*, the agr mgrA double mutant strain; N315 WT, the N315 WT strain; N315Δ*mgrA*, the N315 *mgrA* mutant strain.

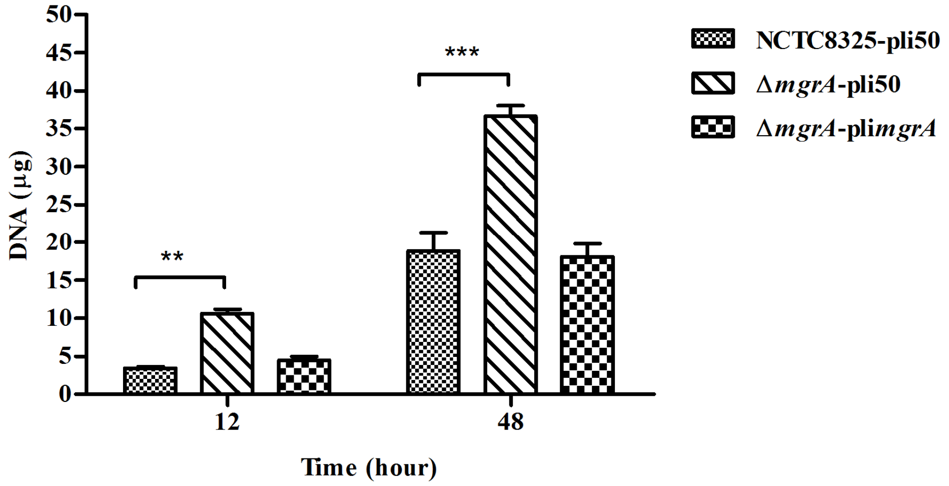

Figure S3. Extracellular DNA (eDNA) levels in biofilms. Static biofilms were grown at 37℃ for 12h or 48h in 24 wells plates containing 1 ml of culture in each well, and biofilms were collected and treated with 10 µg/ml proteinase K in PBS at 37℃ for 2h to release eDNA from biofilms. The samples were extracted once with phenol/chloroform/isoa-myl alcohol (25:24:1), and isopropanol was used to precipitated eDNA. The isopropanol-precipitated DNA was collected through centrifugation for 20 min at 4 °C and 12,000 rpm, washed twice with 75 % (v/v) ethanol, air-dried, and dissolved in 400 µl of water. The quantitation of DNA was measured by ultraviolet spectrophotometry. Values are from three biological replicates ± SEM. Statistical values are determined by Two-way ANOVA, **, $P<0.01$; ***, $P<0.001$. Abbreviations: NCTC8325-pli50, the NCTC8325 WT strain carrying plasmid pli50; Δ*mgrA*-pli50, the *mgrA* mutant strain carrying plasmid pli50; Δ*mgrA*-pli*mgrA*, the *mgrA* mutant strain carrying plasmid pli*mgrA*.

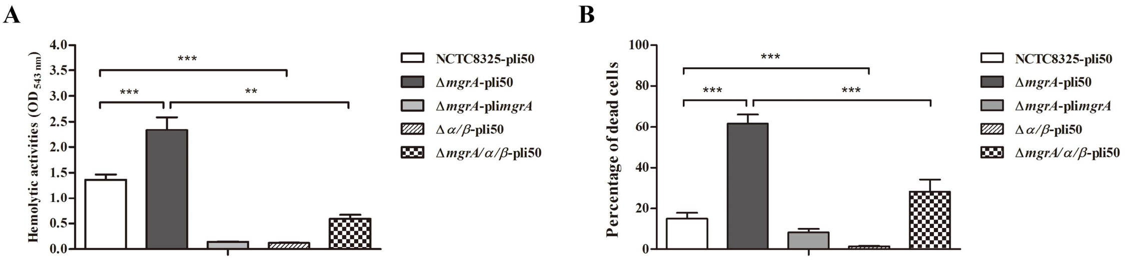

Figure S4. The cell lysis ability of culture filtrates. (A) The ability to lyse blood cells. Hemolytic activity was determined by incubating culture filtrate samples with a 3% (v/v) suspension of sheep red blood cells and incubation at 37℃ for 1h. The filtered bacterial overnight culture supernatants were diluted in PBS and added to sheep erythrocytes at a final concentration of 1:200 (v/v). Values are from three biological replicates ± SEM. Statistical values were determined by One-way ANOVA, **, $P<0.01$; ***, $P<0.001$. (B) The ability to lyse epithelial cell. The 293T cells were incubated in DMEM containing 10% fetal bovine serum at 37℃ with 5% CO_2. The filtered bacterial overnight culture supernatants were diluted in DMEM and added to 293T cells at a final concentration of 1:10 (v/v). Values are from three biological replicates ± SEM. Statistical values were determined by One-way ANOVA, ***, $P<0.001$. Abbreviations: NCTC8325-pli50, the NCTC8325 WT strain carrying plasmid pli50; Δ*mgrA*-pli50, the *mgrA* mutant strain carrying plasmid pli50; Δ*mgrA*-pli*mgrA*, the *mgrA* mutant strain carrying plasmid pli*mgrA*; Δ*α/β*-pli50, the *psmα psmβ* double mutant strain carrying plasmid pli50; Δ*mgrA/α/β*-pli50, the *mgrA psmα psmβ* triple mutant strain carrying plasmid pli50.
